# Effects of Post-Added Alcohol Modifiers on Magnesium Ethoxide Support on Ziegler–Natta Catalyst and Propylene Polymerization Performance

**DOI:** 10.3390/polym18212668

**Published:** 2026-10-28

**Authors:** Hegang Ren, Zhongcheng Chen, Hongyu Chen, Qi Yao, Jingjie Gao, Bo Yang

**Affiliations:** School of Material Science and Engineering, Guangdong University of Petrochemical Technology, Maoming 525000, China; rhg@gdupt.edu.cn (H.R.); yaoqi@gdupt.edu.cn (Q.Y.)

**Keywords:** magnesium ethoxide, alcohol modification, Ziegler–Natta catalyst, pore structure, propylene polymerization

## Abstract

In this work, isopropanol, n-butanol and 2-methoxyethanol were introduced as post-added alcohol modifiers after the preliminary formation of magnesium ethoxide particles. The effects of these alcohol modifiers on the structure of magnesium ethoxide supports, Ziegler–Natta catalysts and propylene polymerization performance were systematically explored. The supports and catalysts were characterized by scanning electron microscopy (SEM), X-ray diffraction (XRD) and N_2_ adsorption–desorption, and the bulk propylene polymerization performances of the resultant catalysts were evaluated. The results demonstrate that alcohol modifiers regulate the nucleation and crystal growth of magnesium ethoxide by altering solvent polarity, steric hindrance and coordination capacity toward magnesium ions. Isopropanol-modified magnesium ethoxide exhibits the smallest particle size. Alcohol modification does not change the crystalline phase of magnesium ethoxide, but adjusts the stacking order and crystallinity of its layered structure. After titanation with TiCl_4_, mesoporous structures are successfully constructed for alcohol-modified catalysts. The catalyst Cat-iPr modified by isopropanol achieves a specific surface area of up to 218.81 m^2^·g^−1^. Alcohol modification tends to refine MgCl_2_ crystallites and introduce structural disorder, as indicated by XRD peak broadening. Although titanium loading decreases slightly upon alcohol modification, the optimized pore structure improves the accessibility of active sites; consequently, all modified catalysts show higher polymerization activity than the unmodified reference catalyst. Isopropanol and n-butanol modification improve the isotactic index, bulk density and crystallization temperature of polypropylene. In contrast, the ether linkage in 2-methoxyethanol may interfere with the stereodirecting coordination, which is plausibly correlated with the reduced isotacticity and crystallization performance of the resulting polypropylene. Alcohol modification appears to facilitate chain-transfer events and decreases the average molecular weight of polypropylene. Isopropanol and n-butanol broaden the molecular-weight distribution, which is beneficial for polymer processability. Owing to the particle replication effect, the polymer products from the modified catalysts contain low fine-powder fractions, satisfying the particle-size requirements for industrial polypropylene production. This alcohol-modification strategy enables directional tuning of the support pore and crystalline structures while preserving macroscopic particle integrity, offering new insights for the development of high-performance magnesium-ethoxide-based Ziegler–Natta catalysts.

## 1. Introduction

Polyolefins, produced via olefin polymerization, are among the most commercially important thermoplastic polymers worldwide. Their broad application scope stems from their balanced material performance and relatively low manufacturing cost. With continuously growing market demand and emerging application scenarios, considerable efforts have been devoted to improving both production efficiency and final polymer properties, in which rational catalyst design plays an irreplaceable role [1,2].

Heterogeneous Ziegler–Natta catalysts still dominate industrial polyolefin manufacturing. Typically, such catalysts consist of a magnesium-based support, chemically immobilized TiCl_4_ active sites, and alkylaluminum cocatalysts. The physicochemical properties of the support largely determine catalyst productivity, stereoselectivity, monomer-comonomer incorporation behavior, and the particle morphology of the polymer products [3,4,5,6,7,8]. Among the various available precursors, magnesium ethoxide (Mg(OEt)_2_) has attracted extensive attention for propylene polymerization Ziegler–Natta catalysts owing to its excellent morphological controllability, high catalytic activity and favorable stereospecificity [9,10,11,12,13,14,15,16,17]. During catalyst fabrication, solid-state chlorination and titanation convert Mg(OEt)_2_ into an active MgCl_2_/TiCl_4_/donor system. A well-known particle-replication effect indicates that the macroscopic particle architecture of the final catalyst is inherited directly from the initial Mg(OEt)_2_ precursor. Accordingly, the particle and crystal design of the catalyst is largely accomplished at the stage of magnesium ethoxide preparation [16,17,18].

Generally, metallic magnesium reacts with ethanol under initiation by halogen additives to precipitate spherical magnesium ethoxide particles, which are assembled from stacked hexagonal plate-shaped nanocrystals. To investigate the control of particle size and morphology of Mg(OEt)_2_, researchers have employed a magnesium-ethanol-iodine reaction system and characterized the products by scanning electron microscopy (SEM), transmission electron microscopy (TEM) and X-ray diffraction (XRD) to elucidate the formation mechanism [19,20,21,22]. In general, iodine first removes the oxide layer to activate the magnesium powder surface, enabling the reaction between magnesium and ethanol to form magnesium ethoxide, which deposits on the surface; the product then detaches as bulk crystals and eventually assembles into quasi-spherical aggregates. The synthesis parameters strongly influence the particle size, particle-size distribution and particle shape of Mg(OEt)_2_. Introducing third-component modifiers is an effective strategy for adjusting crystal stacking and further tuning the pore structure of the resultant Ziegler–Natta catalyst. Numerous studies have modulated the growth of Mg(OEt)_2_ by introducing ternary components into the reaction system, including metal salts, carboxylic acids and esters. It has been observed that even low concentrations of modifiers (e.g., 1.0 mol%) can significantly alter the particulate and crystalline properties of the resulting Mg(OEt)_2_ [23]. Specific metal chlorides, including MnCl_2_, ZnCl_2_ and FeCl_2_, have been reported to increase particle size and promote spheroidization [16,24,25]. In addition, highly polar alcohols such as 2-methoxyethanol reduce the particle size and increase the bulk density [12,26]. A synergistic approach using n-hexane and silicone oil has also been developed to tailor particle characteristics; this strategy enables precise control over particle size (5–30 μm) and bulk density (0.2–0.7 g/cm^3^) [27]. Nevertheless, in most reported cases, modifiers are added simultaneously at the beginning of the precipitation reaction. Such synchronous addition usually interferes with the nucleation-and-growth processes and readily causes fragmentation and destruction of the macroscopic spherical particles, which restricts further performance optimization. To balance particle integrity and pore-modification effects, only limited doses of modifiers can be adopted in conventional protocols. To date, few systematic investigations have focused on post-addition modification, i.e., adding organic modulators “after preliminary particle formation” of magnesium ethoxide [26,28]. Moreover, for different alcohol-type modifiers (aliphatic alcohols versus ether-containing alcohols), the structure–performance chain from the modified Mg(OEt)_2_ precursor, through the MgCl_2_-based catalyst, to the final polypropylene powder remains insufficiently clarified.

In this work, we adopt a post-addition synthetic strategy in which different alcohol modifiers (isopropanol, n-butanol and 2-methoxyethanol) are introduced after the preliminary formation of magnesium ethoxide particles. This method enables the crystallinity and microstructure to be regulated without destroying the precursor macroparticles. Scanning electron microscopy (SEM), X-ray diffraction (XRD) and N_2_ adsorption–desorption are employed to characterize the supports and catalysts, and bulk propylene polymerization tests are performed to evaluate the catalytic performance and the properties of the resulting polymers. We aim to reveal how the steric-hindrance and coordination effects of the different alcohol modifiers regulate precursor crystallization, catalyst pore architecture, the active-site microenvironment, catalyst fragmentation behavior and the ultimate performance of the polypropylene products. This study provides new insights for the custom design of high-performance magnesium-ethoxide-derived Ziegler–Natta catalysts for industrial polypropylene production.

## 2. Experiments

### 2.1. General Procedures and Materials

All manipulations of the air- or moisture-sensitive materials were carried out under a dry argon atmosphere with standard Schlenk techniques. Magnesium powder, with a particle size of 75–100 μm, was purchased from Sinopharm Chemical Reagent Co., Ltd., Shanghai, China. Iodine (I_2_), dibutyl phthalate (DBP), titanium tetrachloride (TiCl_4_) were obtained from Shanghai Titan Scientific Co., Ltd., Shanghai, China, and used as received. Ethanol (EtOH), Isopropanol, 1-butanol, ethylene glycol monomethyl ether, toluene, and n-hexane were purchased from Xilong Scientific Co., Ltd., Shantou, China, and dried with a 4 Å molecular sieve. Triethylaluminium (TEA, 1 M heptane solution) was purchased from Aldrich and applied without further purification. Propylene of polymerization grade (kindly supplied by ZhongKe (Guangdong) Refinery &Petrochemical Co., Ltd., Zhanjiang, China) was used as received.

### 2.2. Magnesium Ethoxide Synthesis

Four magnesium ethoxide samples (designated MGE-A to MGE-D) were prepared by reacting metallic magnesium with ethanol in the presence of iodine. The synthesis was carried out in a 500 mL glass Schlenk reactor fitted with a mechanical stirrer and maintained under a nitrogen atmosphere. In a typical procedure, iodine (0.68 g) was first dissolved in ethanol (32 mL); the solution was then heated to 70 °C under continuous stirring. Subsequently, magnesium powder (24 g in total) was introduced in eight equal portions at 10 min intervals, together with the required amount of ethanol. After the final portion had been added, the organic modifier was charged into the reactor. The resulting mixture was maintained at 70 °C for 3 h with continuous stirring. Finally, the slurry was cooled to 50 °C, and the solid product was collected by filtration and dried under vacuum.

### 2.3. Catalyst Synthesis

A 500 mL Schlenk reactor equipped with a mechanical stirrer was charged with 15 g of Mg(OEt)_2_ and 150 mL of toluene. The resulting suspension was cooled slowly to −20 °C in an ice–salt bath, and 30 mL of TiCl_4_ was then added dropwise with stirring. The mixture was subsequently heated to 70 °C over 4 h, after which a calculated amount of DBP (Mg(OEt)_2_/DBP molar ratio = 0.15) was introduced. The temperature was then raised to 110 °C and held for 2 h. After the first titanium loading step, unreacted TiCl_4_, toluene and the formed inactive titanium alkoxy species were removed from the system under vacuum. A further 30 mL of TiCl_4_ and 150 mL of toluene were then added, and the mixture was maintained at 110 °C for another 2 h. The liquid components were again removed by vacuum stripping. Subsequently, an additional 30 mL of TiCl_4_ and 150 mL of toluene were introduced, and the reaction was continued at 110 °C for 1 h. The solid product was separated by filtration and washed sequentially: twice with toluene at 100 °C and five times with n-hexane at 60 °C. After vacuum drying, the final catalysts were obtained and denoted as Cat-Et (from MGE-A), Cat-*i*Pr (from MGE-B), Cat-*n*Bu (from MGE-C) and Cat-ME (from MGE-D).

### 2.4. Propylene Bulk Polymerization

Propylene polymerization was carried out in a 2 L autoclave fitted with a mechanical stirrer operated at 200 rpm. Prior to the reaction, the autoclave was purged twice with dry nitrogen and once with propylene. Under a nitrogen flow, the following components were then charged into the reactor: n-heptane solutions of TEA (3.0 mmol) and the silane compound (0.15 mmol), the solid catalyst (10 mg), hydrogen (0.05 MPa) and liquefied propylene (500 g). The autoclave was subsequently heated to 70 °C within a few minutes, and the main polymerization was allowed to proceed at this temperature for 60 min. The resulting polymer was recovered and dried under vacuum at 60 °C for 6 h. The catalyst activity was calculated as the mass of polypropylene produced (kg) per unit mass of catalyst used (g).

### 2.5. Characterizations

#### 2.5.1. Mg(OEt)_2_ and Catalyst Characterization

Particle morphology of the Mg(OEt)_2_ samples and the resulting catalysts was examined with a Regulus 8220 scanning electron microscope (SEM; Hitachi High-Tech, Tokyo, Japan). Before examination, the samples were spread evenly onto conductive adhesive tape and sputter-coated with gold for 15 s to improve surface conductivity. For quantitative morphological assessment, more than 200 particles were analyzed from the SEM images using Nano Measurer 1.2 and ImageJ software v1.8.0. D50 is defined as the particle diameter at which the cumulative number distribution reaches 50%, while circularity, expressed as Circularity = 4π × Area/(Perimeter)^2^, was used to describe how closely the particles approach a perfectly circular shape. The bulk density of the Mg(OEt)_2_ powder was obtained by weighing a fixed volume of the powder, essentially following the procedure of Chinese Standard GB/T 23771-2009 [29]. Nitrogen adsorption–desorption measurements were carried out on a BELSORP MINI II instrument to determine the surface areas of the synthesized Mg(OEt)_2_ and catalysts. Specific surface areas (S.A., m^2^/g) were calculated according to the Brunauer–Emmett–Teller (BET) theory, and the total pore volume (V_pore, cm^3^/g) and average pore diameter (d_pore, nm) were derived from d_pore = 4V_pore/S_pore. Pore size distributions were obtained by the Barrett–Joyner–Halenda (BJH) method. The titanium contents of the catalysts were determined photometrically at 410 nm as peroxide complexes using an Analytik Jena Specord UV/Vis spectrophotometer. The crystalline structures of the Mg(OEt)_2_ and catalysts were characterized by X-ray powder diffraction (XRD; Ultima IV, Rigaku Co., Ltd., Tokyo, Japan) with Cu Kα radiation at 40 kV and 30 mA, scanning from 5 to 70° with a step of 0.02° and a rate of 10°·min^−1^. The samples were mounted in a glass holder under a N_2_ atmosphere and covered with a polyethylene film to prevent moisture contact.

#### 2.5.2. Polymer Characterization

The isotactic index (I.I.) of the polypropylene samples was measured by n-heptane extraction. In a typical procedure, 2 g of polypropylene was transferred to a Soxhlet thimble and extracted with boiling n-heptane for 12 h to remove the amorphous fraction; the percentage of polymer remaining after drying was taken as the I.I. Molecular weights and molecular weight distributions (dispersity, Ð) were obtained with a PL 220 gel permeation chromatograph (GPC; Polymer Laboratories, Shropshire, UK) fitted with a differential refractive index detector, a viscometric detector and PL-GEL 10 μm MIXED-B columns (300 × 7.5 mm). GPC measurements were run at 150 °C with 1,2,4-trichlorobenzene as the eluent, and the system was calibrated against polystyrene standards covering a molecular weight range of 600 to 3 × 10^6^ g/mol. Melting (Tm) and crystallization (Tc) temperatures were recorded by differential scanning calorimetry (DSC) using a DSC 214 instrument (Netzsch GmbH, Selb, Germany). Approximately 5 mg of polypropylene film was encapsulated in an aluminum pan and heated from 30 to 200 °C at 10 °C/min, held to erase the thermal history, and then cooled to room temperature at the same rate; a second heating scan to 200 °C at 10 °C/min was used to obtain the melting endotherm and Tm. The bulk density of the polypropylene was measured according to ISO 60:2023 [30].

## 3. Results and Discussion

### 3.1. Synthesis and Characterization of Magnesium Ethoxide Supports

**Figure 1:**
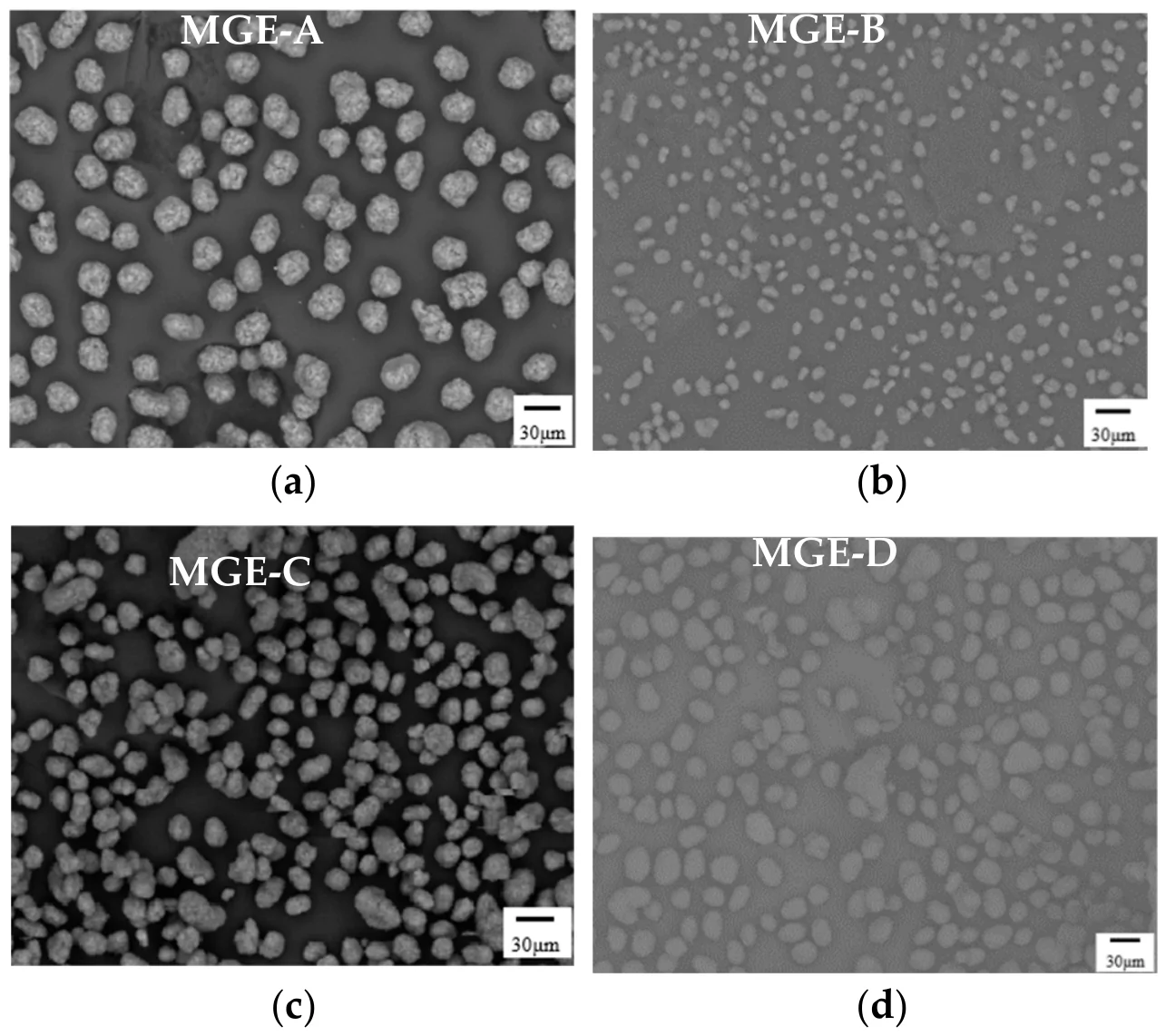
SEM images of Mg(OEt)_2_ particles: (**a**) MGE-A (×300), (**b**) MGE-B (×300), (**c**) MGE-C (×300), (**d**) MGE-D (×300).

Four types of Mg(OEt)_2_ particles were synthesized using different alcohol raw materials, whose properties are summarized in Table 1. Representative SEM images are shown in Figure 1. As shown in Table 1, without the addition of alcohol modifiers, the magnesium ethoxide support (MGE-A) exhibits large, loosely packed particles with a low bulk density. During the synthesis of the magnesium ethoxide support, the introduction of a small amount of alcohol modifier significantly reduces the particle size of the support, increases its bulk density and affects its sphericity. When isopropanol is used as the modifier, the resulting magnesium ethoxide support (MGE-B) has the smallest particle size, with an average particle diameter of 9.43 μm. Compared with MGE-A, the particle size of MGE-B decreases by approximately 62%, and its bulk density shows the most prominent increase, while the sphericity remains unchanged. For the supports synthesized with n-butanol or 2-methoxyethanol, the average particle sizes are markedly reduced, and the bulk densities are improved relative to MGE-A. However, the particle sphericity deteriorates, and the extent of particle size reduction is less significant than that obtained with isopropanol. This may be because the addition of alcohol modifiers alters the solvent polarity, viscosity or coordination ability toward magnesium ions, thereby affecting the nucleation rate and crystal growth [26]. Isopropanol is most favorable for rapid nucleation, while its steric hindrance suppresses crystal growth [13], resulting in the smallest and most compact particles. In contrast, 2-methoxyethanol contains ether oxygen bonds, which may form strong complexes with magnesium ions; this hinders nucleation or leads to uneven crystal growth and consequently to a slight reduction in particle sphericity.

**Figure 2:**
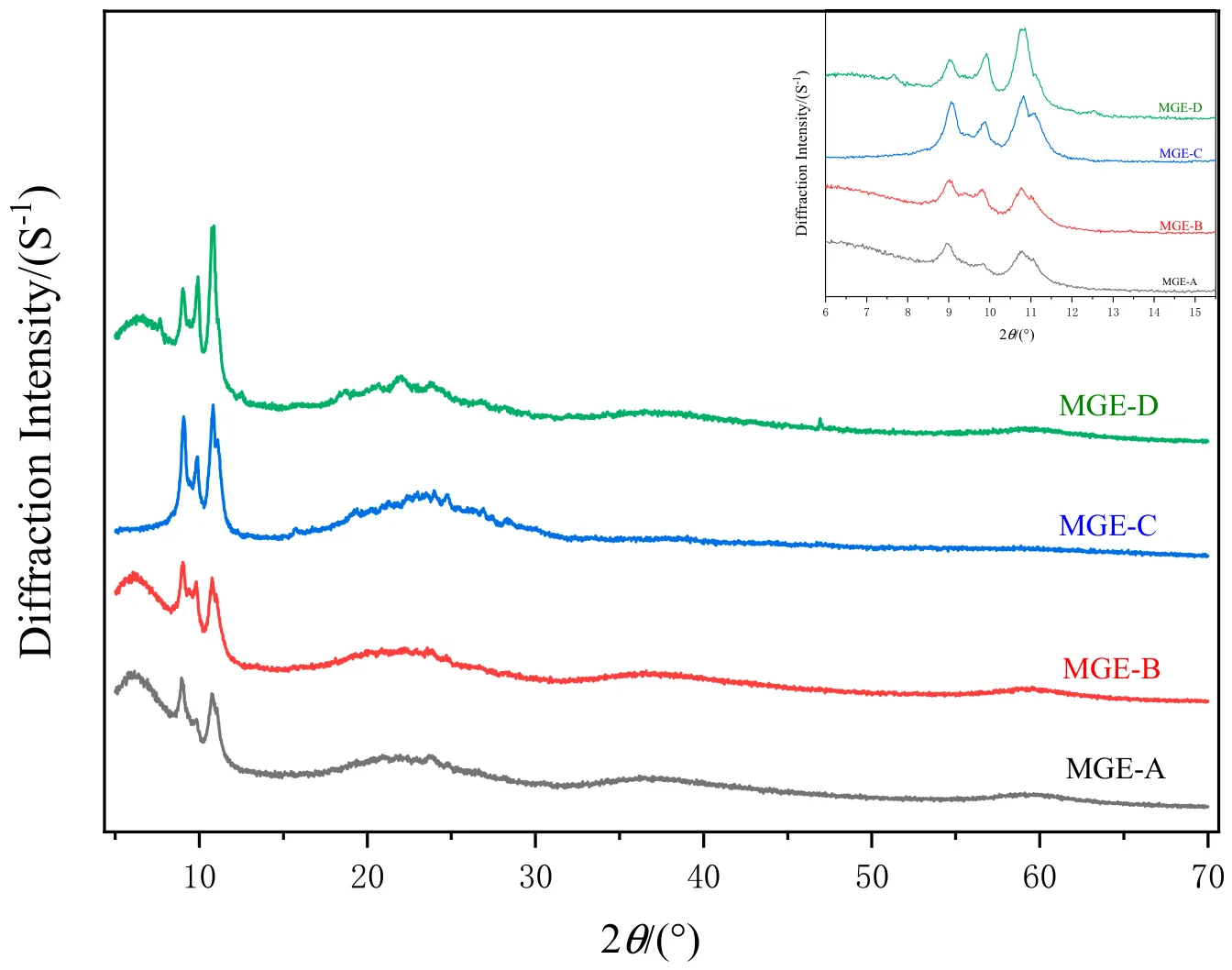
XRD patterns of different Mg(OEt)_2_ particles.

X-ray diffraction (XRD) is a core characterization method in materials science for analyzing crystal structures and phase compositions. Figure 2 shows the XRD patterns of the different magnesium ethoxide supports. All four supports exhibit distinct characteristic diffraction peaks in the low-angle region (2θ < 15°), indicating that the samples possess a typical layered crystal structure. No obvious sharp impurity peaks are observed for any of the samples in the high-angle region (2θ > 20°); only broad diffuse peaks appear. Combined with the quasi-spherical morphology of all four supports shown in Figure 2, it can be concluded that the introduction of alcohol modifiers does not change the overall crystalline phase composition of magnesium ethoxide, but only alters the packing order and crystallinity of the particles.

As can be seen from Figure 2, the MGE-A support shows the most prominent peak broadening in the high-angle region, together with the weakest intensity and the broadest shape of the low-angle characteristic peak. This further demonstrates that MGE-A has the poorest internal crystallinity and the most disordered arrangement of layered structures, resulting in the weakest diffraction signal. The MGE-B support presents a relatively weak intensity and a slightly broadened shape of the low-angle characteristic peak, suggesting that the addition of isopropanol strongly inhibits particle growth and refines the crystallite size [13]. The MGE-C support exhibits a relatively high intensity of the low-angle characteristic peak, indicating that the support particles obtained with n-butanol possess good crystallinity, with the regularity of the layered structures being second only to that of MGE-D. The MGE-D support exhibits the highest and sharpest low-angle characteristic peak, revealing that the addition of 2-methoxyethanol promotes oriented particle growth and perfect crystallization, achieving the best long-range order of the layered structures and thus the highest diffraction peak intensity. This is attributed to the strongest polarity of 2-methoxyethanol. These results verify that the polarity and steric hindrance of the alcohol modifiers can regulate the “refinement and crystallization” of the magnesium ethoxide support particles.

### 3.2. Preparation and Characterization of Mg(OEt)_2_-Derived Ziegler–Natta Catalysts

The pore-structure parameters and titanium contents of the catalysts derived from the different Mg(OEt)_2_ precursors are summarized in Table 2. The unmodified Cat-Et is dense, with an extremely low specific surface area and pore volume. Modification with isopropanol or 2-methoxyethanol is highly effective for pore construction. Cat-iPr achieves the maximum BET surface area of 218.81 m^2^·g^−1^ and a pore volume of 0.21 cm^3^·g^−1^, but the minimum pore size. Cat-ME exhibits a pore volume comparable to that of Cat-iPr and a slightly lower specific surface area. Modification with n-butanol results in only limited improvement in the pore structure. The steric hindrance and coordination capability of the alcohol modifiers are key factors governing pore-structure tuning. Branched isopropanol restrains crystallite growth through its large steric hindrance; 2-methoxyethanol provides dual coordination through its hydroxyl and ether bonds; and long-chain n-butanol shows a weak coordination ability. Alcohol modification slightly reduces the titanium loading; however, it is the difference in pore architecture, rather than the variation in titanium content, that dominates active-site exposure and monomer mass transfer. The differences in pore structure further alter the dispersion state of the Ti active species and affect monomer diffusion, the stereoselectivity and the bulk density of the resulting polypropylene.

**Figure 3:**
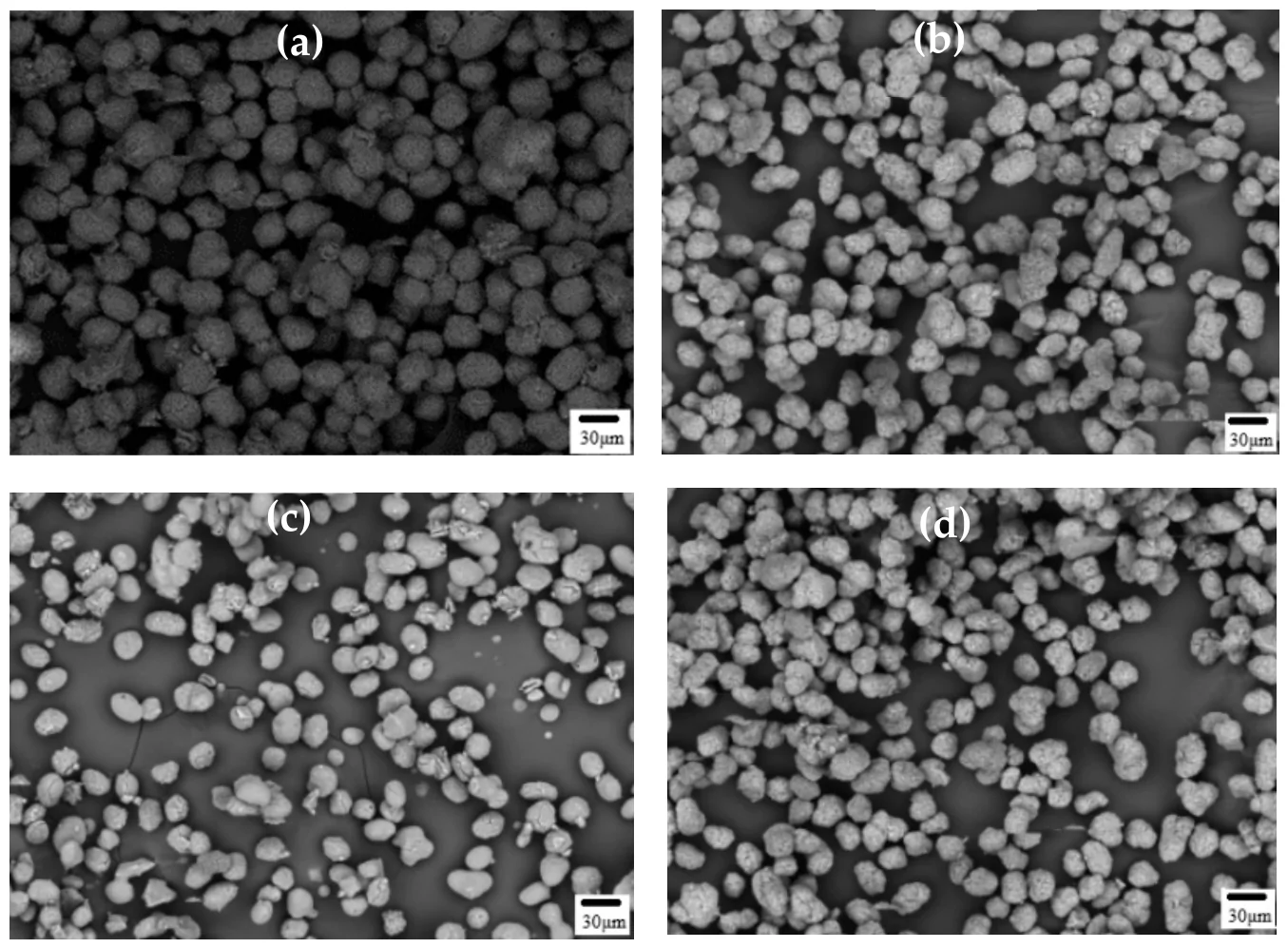
SEM images of the four catalysts: (**a**) Cat-Et (×300), (**b**) Cat-*i*Pr (×300), (**c**) Cat-*n*Bu (×300), (**d**) Cat-ME (×300).

Figure 3 shows the SEM images of four magnesium ethoxide-based Ziegler–Natta catalysts. All catalyst particles exhibit quasi-spherical shapes and well replicate the morphology of the parent magnesium ethoxide support, demonstrating that alcohol modification and subsequent titanation largely preserve the original particle outline of the support. This is the typical replication effect for Mg(OEt)_2_-type Ziegler–Natta catalysts.

The unmodified Cat-Et shows a larger average particle size with plumper particle contours. After alcohol modification, Cat-*i*Pr (isopropanol-modified), Cat-*n*Bu (n-butanol-modified) and Cat-ME (2-methoxyethanol-modified) exhibit decreased average particle sizes. Cat-*i*Pr presents superior particle size uniformity, while a small amount of irregular particles and slight inter-particle adhesion are observed in Cat-*n*Bu and Cat-ME. Differences in particle morphology and size distribution affect the pore structure and dispersion of Ti active species, further modulating monomer mass transfer during polymerization and tuning the morphology and bulk density of polymer powder. This trend can be supported by the subsequent polymerization results (Table 3 and Table 4).

**Figure 4:**
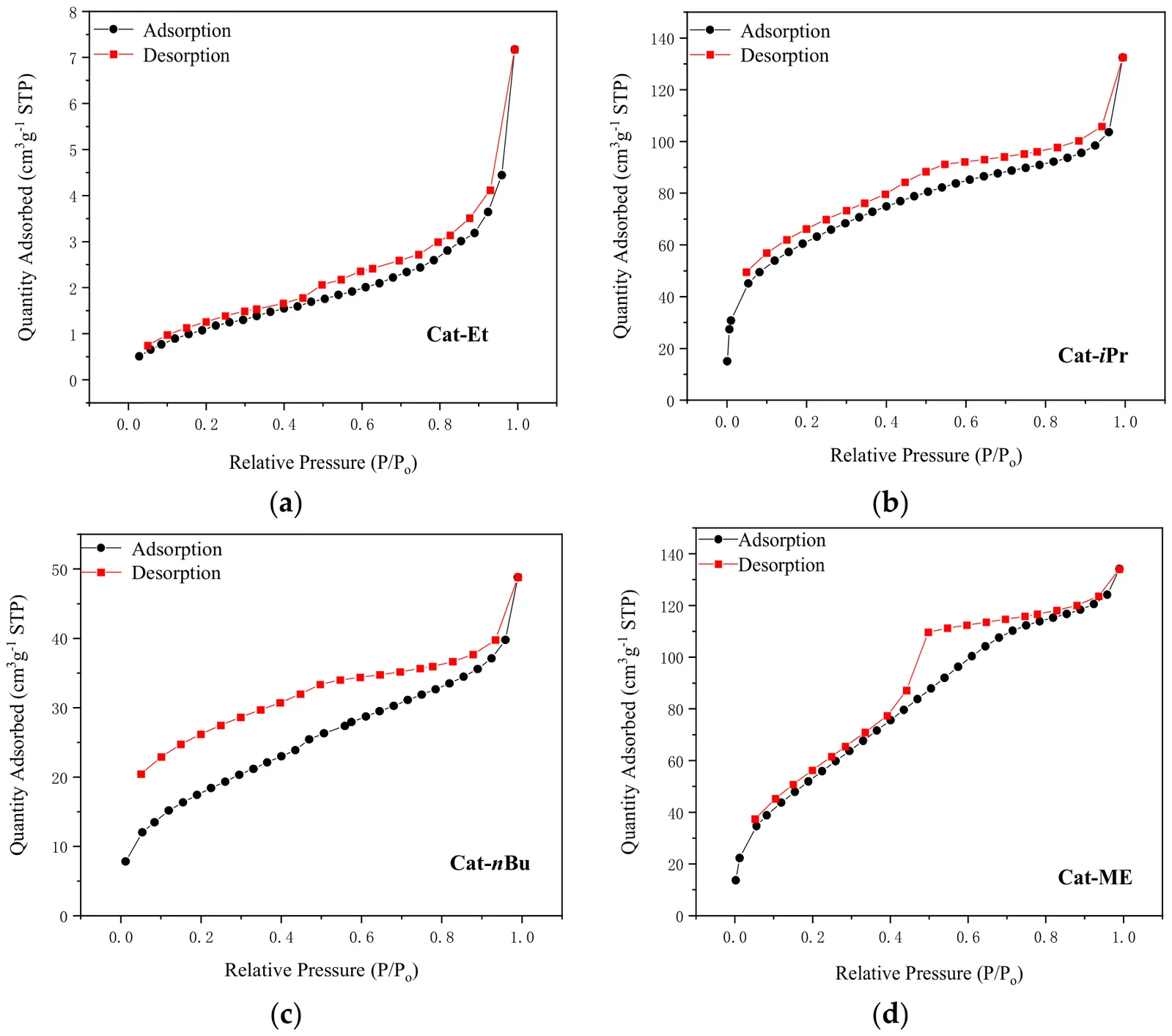
N_2_ adsorption/desorption isotherms for catalyst samples: (**a**) Cat-Et, (**b**) Cat-*i*Pr, (**c**) Cat-*n*Bu, (**d**) Cat-ME.

Figure 4 shows the N_2_ adsorption–desorption isotherms and pore-size distribution curves of the four catalyst microspheres. According to the BET results and the N_2_ adsorption–desorption isotherms, the Cat-Et sample prepared with ethanol alone exhibits an extremely low specific surface area of only 4.40 m^2^·g^−1^ and a negligible pore volume. Its adsorption–desorption curve shows no obvious hysteresis loop, which is characteristic of a dense macroporous material. After the introduction of isopropanol, n-butanol or 2-methoxyethanol as alcohol modifiers, all the catalysts display type-IV isotherms together with typical mesoporous hysteresis loops. These features indicate that the alcohol modifiers favor the formation of mesoporous structures, which may be associated with a moderation of the crystallization behavior of the MgCl_2_ support and suppression of crystallite agglomeration.

The XRD results show that all catalysts exhibit characteristic diffraction peaks assigned to δ-MgCl_2_ at 2θ ≈ 16.7–16.9°, 30.8–31.1° and 50.2°, indicating that alcohol modification does not alter the main crystalline phase of MgCl_2_. The unmodified Cat-Et gives relatively sharp diffraction peaks corresponding to larger MgCl_2_ crystallites and higher crystallinity. After alcohol modification, the MgCl_2_ diffraction peaks become broadened and their intensity decreases, which indicates that alcohol coordination restrains MgCl_2_ grain growth and generates smaller crystallites. Peak broadening and reduced peak intensity may reflect a decrease in crystallite size and/or an increase in structural disorder (microstrain), rather than a direct increase in crystal defects. Cat-iPr shows the most prominent peak-broadening effect, consistent with its highest BET specific surface area. No new crystalline diffraction peaks emerge in the XRD patterns, indicating that the alcohol modifiers function through coordination interactions with MgCl_2_ rather than by forming independent crystalline phases. The modified catalysts exhibit merged broad peaks within 28–35° (2θ), representing a higher degree of stacking disorder. The changes in the crystal structure of the original Mg(OEt)_2_ precursors induced by the different modifiers are inherited by the MgCl_2_ phase generated in the catalysts.

### 3.3. Propylene Bulk Polymerization Performance and Polypropylene Properties

The Ziegler–Natta catalysts tuned by the alcohol-modified magnesium ethoxide supports exhibit differentiated propylene polymerization performances, as listed in Table 3. As shown in the table, all the modified catalysts possess higher polymerization activity than the reference Cat-Et (18.50 kg PP·g^−1^ Cat): Cat-iPr delivers an activity of 20.18 kg PP·g^−1^ Cat, Cat-nBu shows the maximum activity of 21.62 kg PP·g^−1^ Cat, and Cat-ME reaches 21.00 kg PP·g^−1^ Cat. These results indicate that modifying the support with alcohol modifiers optimizes its pore structure and improves the accessibility of active sites, which counteracts the adverse effect caused by the decreased titanium loading. In addition, although Cat-iPr has the highest specific surface area, its excessively small average pore size increases the mass-transfer resistance of the propylene monomer, so that part of the active sites inside the catalyst particles cannot be fully utilized. Despite its well-developed pore structure, Cat-ME features the lowest titanium loading, and its ether linkages may disturb the coordination environment of the active centers. Cat-nBu achieves an optimal balance between specific surface area and pore size. Its larger mesopore size facilitates the diffusion of the propylene monomer into the interior of the catalyst particles and leads to a high utilization efficiency of the active sites. Meanwhile, n-butanol does not introduce ether-oxygen groups that would impair the active centers, and the intrinsic activity of the titanium active species is well retained. Although its titanium loading is lower than that of the reference catalyst, Cat-nBu has the largest number of accessible active centers, and therefore exhibits the highest propylene polymerization activity.

**Figure 5:**
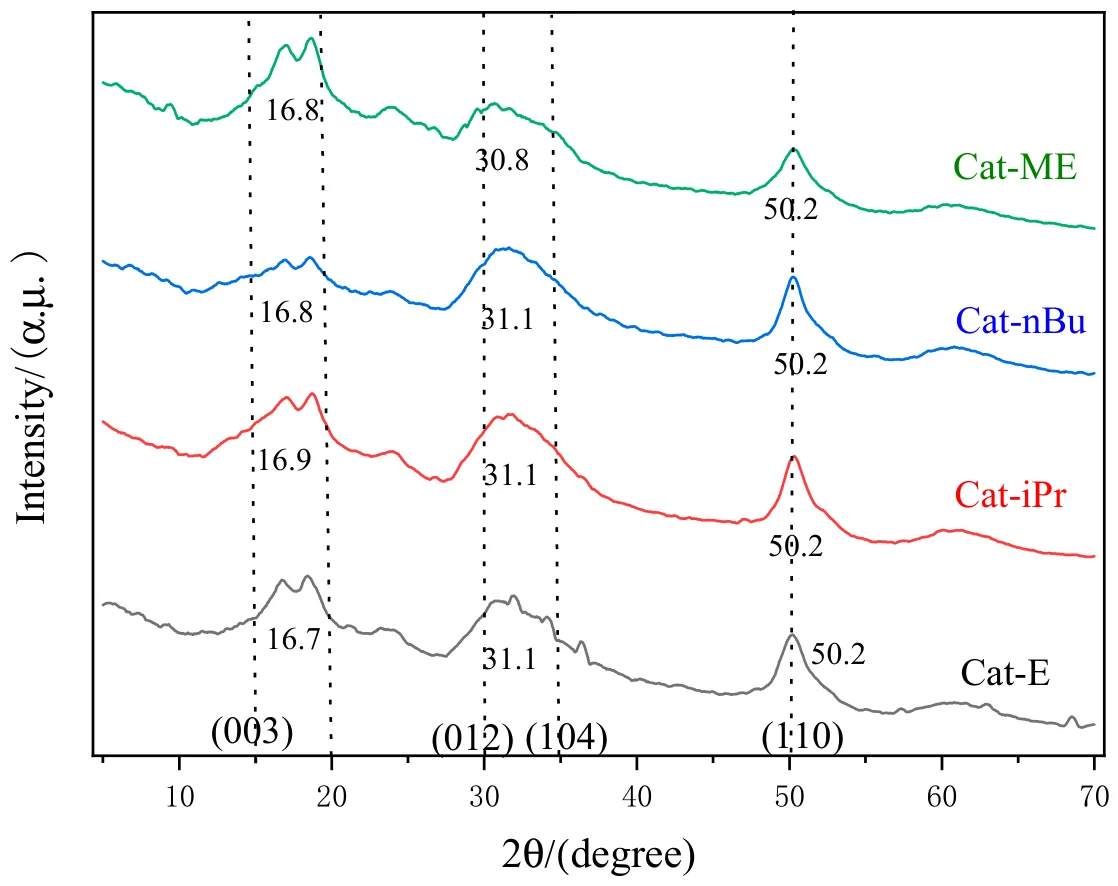
XRD patterns of the four catalysts.

It can also be seen from the table that the bulk density (BD) of polypropylene essentially reflects the packing compactness of the powder particles. The BD of the polypropylene obtained with the reference Cat-Et is only 0.38 g·cm^−3^; after modification with the alcohol modifiers, the bulk density of the polypropylene increases significantly, and the product from Cat-iPr reaches a bulk density of 0.48 g·cm^−3^. The underlying reasons are as follows. The unmodified Cat-Et is prepared from loose MGE-A, and the resulting catalyst particles are dense, with almost no mesopores, and suffer from severe crystallite agglomeration. Uncontrollable catalyst fragmentation occurs during polymerization, readily generating a small number of oversized and irregular particles with large voids among the powder particles (see the optical photographs of the polypropylene particles in Figure 5), which consequently leads to a low bulk density of the polypropylene powder. By contrast, during the titanation of the alcohol-modified Mg(OEt)_2_, the coordination effect of the alcohol suppresses the agglomeration of MgCl_2_ crystallites and constructs abundant uniform mesopores with refined MgCl_2_ crystallites. The catalyst retains intact spherical particles, which are mesoporous microspheres assembled from numerous nano-sized MgCl_2_ crystallites. During propylene polymerization, the mesoporous catalyst enables uniform monomer mass transfer and undergoes controllable particle fragmentation. Relying on the support-to-catalyst-to-polymer particle replication effect, a polypropylene powder with high sphericity, a narrow particle-size distribution and few fragment-like particles is obtained. The voids between particles are reduced, which ultimately improves the bulk density of the polypropylene. Specifically, the isopropanol-modified catalyst possesses a small particle size, high sphericity and a uniform pore size, and exhibits a moderate degree of fragmentation during polymerization, producing quasi-spherical polypropylene particles with a concentrated size distribution and therefore the highest bulk density. Overall, these results indicate that tailoring the support pore structure through alcohol modification is an effective route to control the fragmentation behavior and the final powder morphology of polypropylene.

It can also be observed from the above table that the different modifiers exert remarkably different regulatory effects on the stereoselectivity of the catalysts. The isotactic index of the polypropylene obtained with the unmodified reference catalyst Cat-Et is 98.42%. After modification with isopropanol and n-butanol, the stereoselectivity of the catalysts is greatly improved, and the isotactic indices of the corresponding polypropylene samples rise to 99.12% and 99.10%, respectively, both higher than that of the blank sample. This is because the alcohol modifiers can optimize the micromorphology and pore structure of the MgCl_2_ support, tune the microenvironment of the catalyst active sites, and thereby enhance the stereodirecting capability of the active sites, reducing the content of the atactic polypropylene fraction and increasing the isotacticity of the products. Among them, the isopropanol-modified Cat-*i*Pr exhibits the optimal stereoselectivity. In contrast, Cat-ME modified with 2-methoxyethanol shows the opposite tendency, and the isotactic index of its resulting polypropylene drops to 98.03%, slightly lower than that of the blank sample. This indicates that the ether-bond structure in 2-methoxyethanol may interfere with the stereodirecting coordination ability of the catalyst active sites and is unfavorable for the generation of stereoregular polypropylene chains.

**Figure 6:**
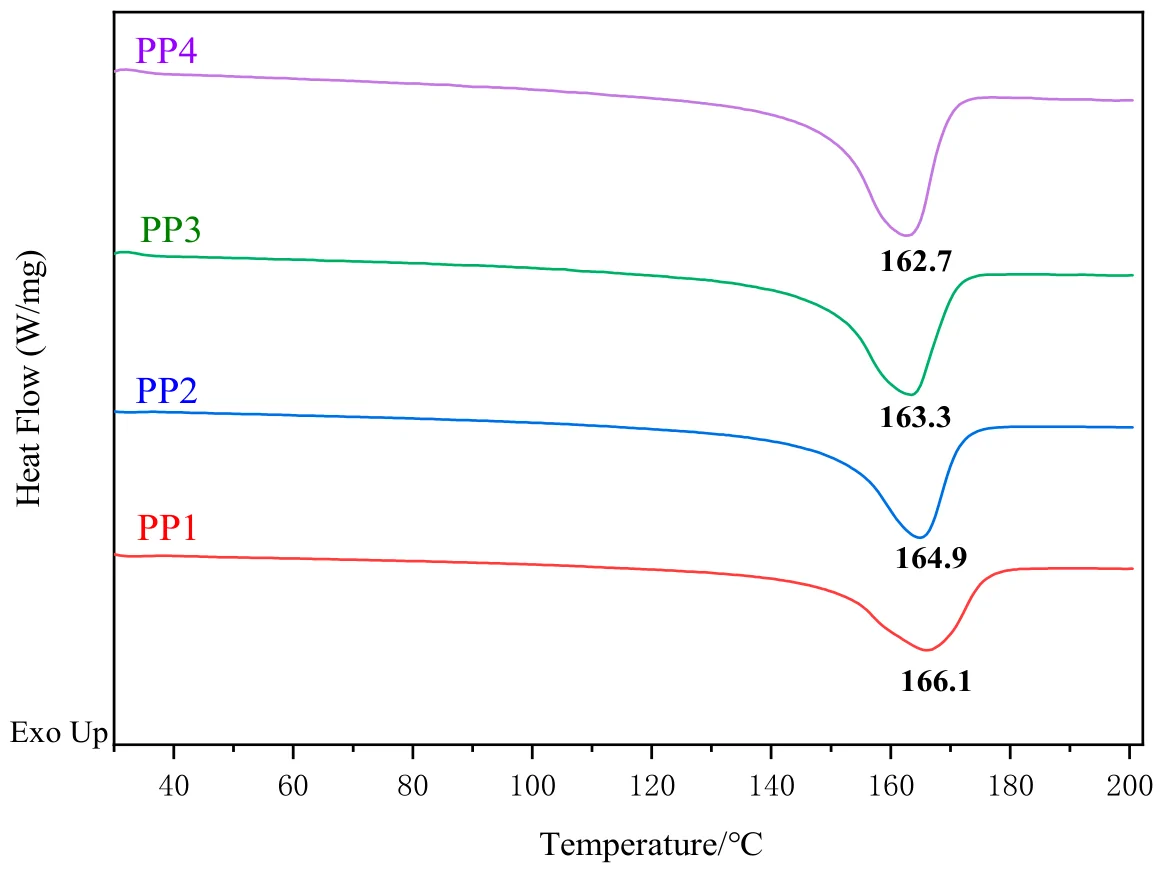
DSC curves of various polypropylene samples.

In addition, the melting temperature (*T*_m_) of the polypropylene from the reference Cat-Et is 166.10 °C. The melting temperatures of the polypropylene decrease to varying degrees after the introduction of the modifiers, among which Cat-ME gives the lowest *T*_m_ of only 162.70 °C. This variation trend is consistent with that of the isotactic index. The crystallization temperature (*T*_c_) characterizes the nucleation and crystallization capacity of the polymers upon cooling; a higher *T*_c_ corresponds to a faster crystallization rate. The crystallization temperatures of the polypropylene from Cat-*i*Pr and Cat-nBu are 103.70 °C and 104.40 °C, respectively, both higher than the 100.00 °C of the reference sample. This indicates that the polypropylene prepared with the isopropanol- and n-butanol-modified catalysts possesses a stronger nucleation capacity and higher crystal perfection, which is consistent with the observed trend in isotacticity. In contrast, the *T*_c_ of the Cat-ME sample decreases sharply to 93.90 °C, with an obviously weakened crystallization ability, which further supports its lower isotacticity. The higher *T*_c_ values of the Cat-*i*Pr and Cat-*n*Bu products are consistent with their higher isotactic indices, indicating that the modified catalysts favor the formation of more stereoregular chains that crystallize more readily. The DSC curves of the different polypropylene samples are shown in Figure 6.

**Figure 7:**
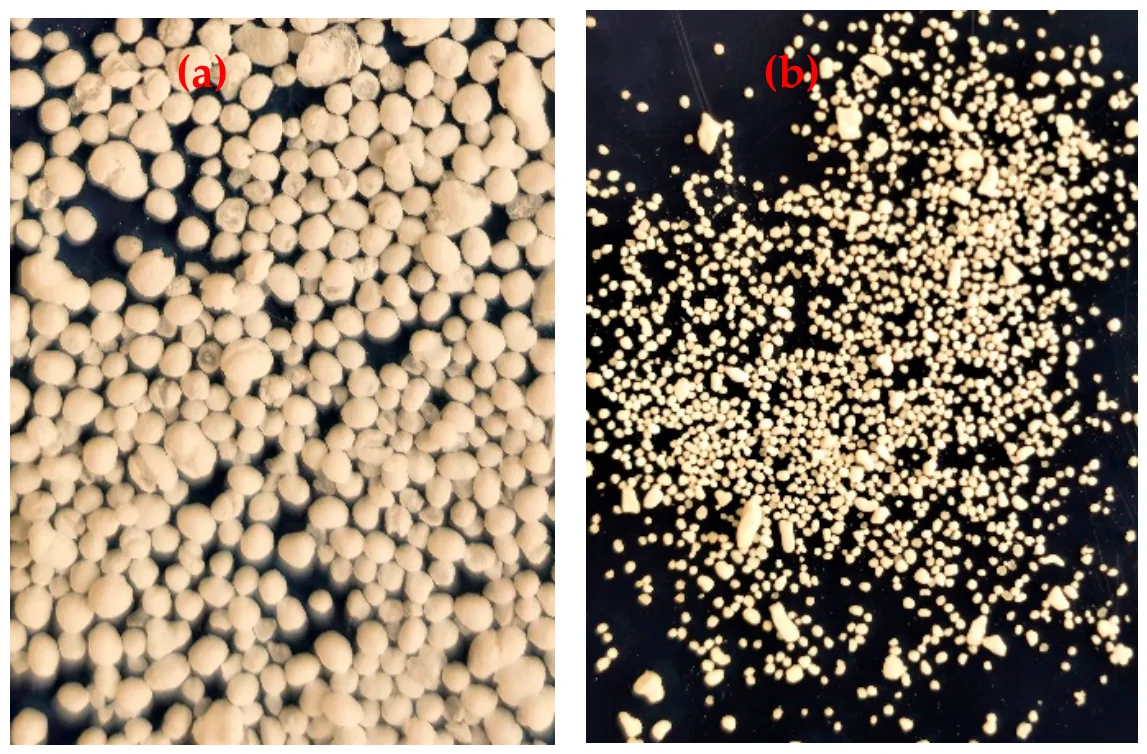

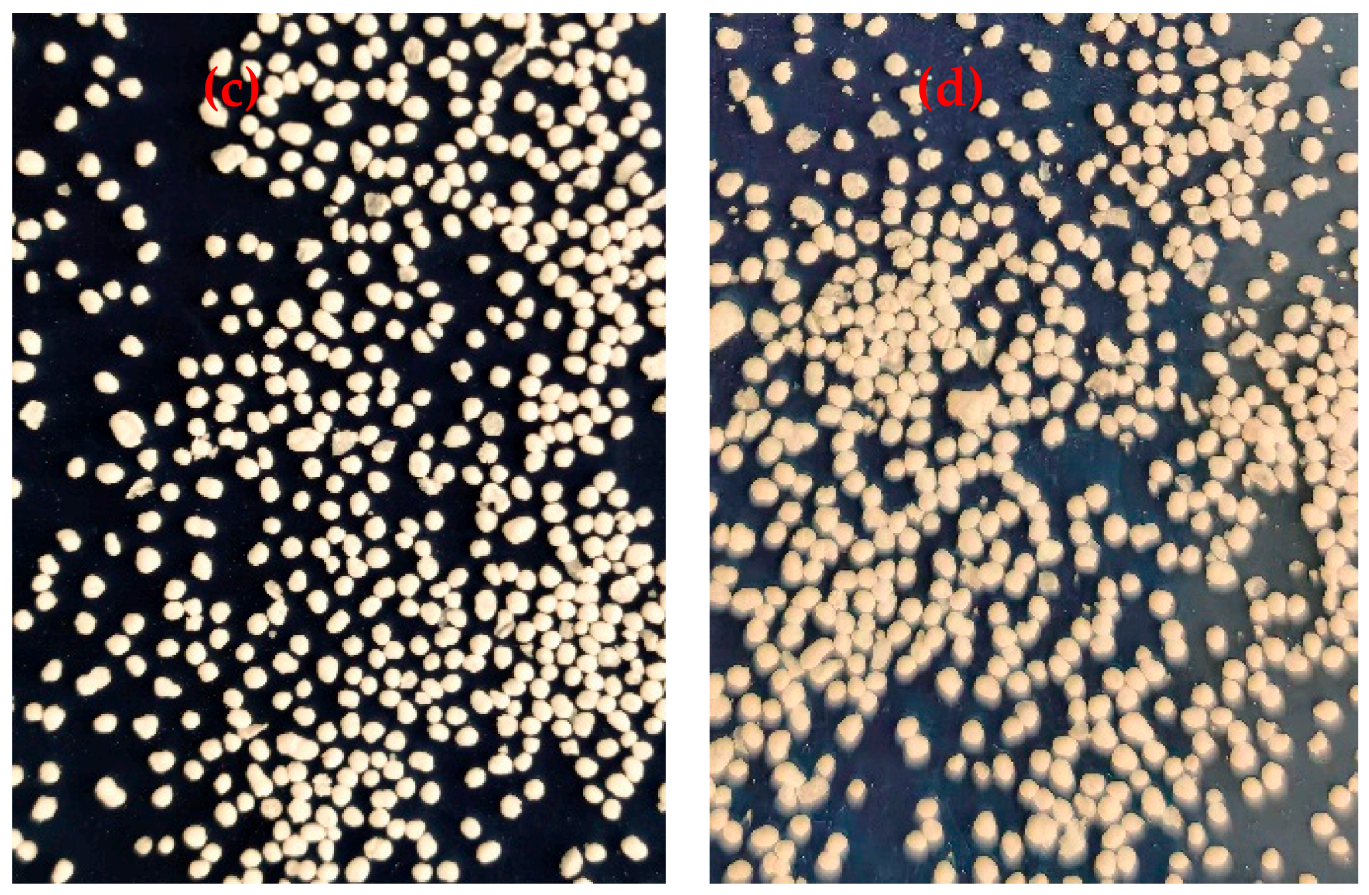
Photograph of polypropylene synthesized with Cat-Et (**a**), Cat-iPr (**b**), Cat-nBu (**c**) and Cat-ME (**d**).

Table 4 summarizes the particle-size distribution results for the polypropylene powders prepared with the different catalysts, and optical photographs of the as-synthesized polypropylene powders are presented in Figure 7. The particle-size distribution data of the polypropylene powders show that the pore structure of the supports tuned by alcohol modification significantly alters the particle fragmentation behavior of the catalysts during propylene polymerization, which is consistent with the particle replication effect. The PP1 powder produced with the unmodified Cat-Et is dominated by large particles, with the fraction of particles larger than 833 μm exceeding 94 wt%. Upon alcohol modification, well-developed pore structures are formed in the supports. Accordingly, the catalysts tend to fragment more easily during polymerization, and the overall particle size of the resulting polymer powders decreases. PP2 from Cat-iPr exhibits the smallest particle size, with its main peak falling in the range of 250–429 μm, which is consistent with a more controllable fragmentation of the catalyst in the case of supports with a high specific surface area and a small pore size. The main particle-size peaks of PP3 (Cat-nBu) and PP4 (Cat-ME) both lie within 429–833 μm. In particular, PP4 obtained from Cat-ME contains more particles in the range of 833–1500 μm. This may be because its larger support pore size is expected to enhance the mechanical strength of the catalyst particles and suppress excessive fragmentation during polymerization. The fine-powder fraction (<125 μm) remains at a low level for all the polypropylene samples, indicating that this series of catalysts possesses excellent particle-morphology stability and can meet the technical requirements of powder particle size for industrial polypropylene production. These results indicate that tuning the support pore structure is an effective strategy for controlling the fragmentation behavior and hence the final powder morphology of polypropylene.

**Figure 8:**
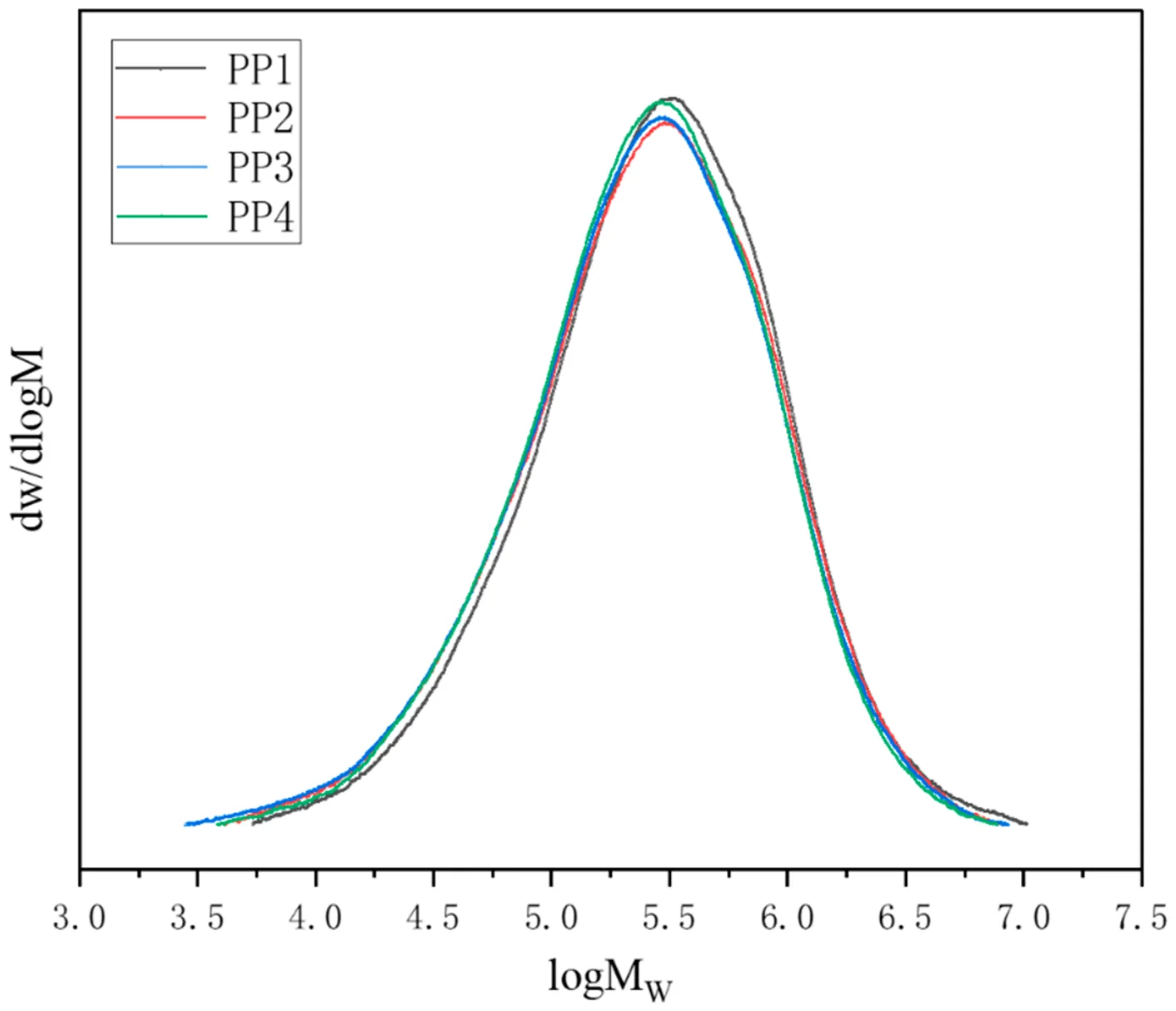
Molecular weight distribution of polymers.

The GPC curve for polypropylene is shown in Figure 8. Table 5 lists the molecular weights and molecular weight distributions of the polypropylene resins synthesized with the different catalysts. Compared with PP1 obtained from the reference Cat-Et, PP2, PP3 and PP4 prepared with the alcohol-modified catalysts exhibit reduced weight-average molecular weight (*M*_w_), number-average molecular weight (*M*_n_), peak molecular weight (*M*_p_) and z-average molecular weight (*M*_z_). PP1 possesses the highest *M_w_* of 5.30 × 10^5^ g·mol^−1^, whereas PP4 shows the lowest *M*_w_ of 4.65 × 10^5^ g·mol^−1^. This indicates that the active sites of the unmodified catalyst are more favorable for continuous molecular-chain growth, with a low probability of chain termination. Among the modified systems, the n-butanol-modified sample exhibits a relatively higher *M*_w_, the isopropanol-modified sample shows the lowest *M*_n_, and the 2-methoxyethanol-modified sample possesses the overall lowest molecular weight. These observations are consistent with the organic modifiers coordinating with the MgCl_2_ support and altering the microenvironment of the active centers, which may in turn promote chain-transfer reactions and accelerate molecular-chain termination, thereby reducing the average molecular-chain length of the polymers. However, direct spectroscopic or kinetic evidence for this mechanism was not obtained in the present work.

Meanwhile, *M*_z_ is highly sensitive to the ultra-high-molecular-weight fractions in polymers. The *M*_z_ of PP1 reaches 15.47 × 10^5^ g·mol^−1^, which is remarkably higher than those of the three alcohol-modified samples. This suggests that the polymerization system of the reference catalyst Cat-Et is prone to generating ultra-high-molecular-weight polypropylene chains. Upon the introduction of the alcohol modifiers, the pore structure of the support is changed, which may in turn modify the microenvironment of the active centers. This may increase the probability of chain-transfer reactions, thereby reducing the molecular-chain length of the polymers and the proportion of ultra-high-molecular-weight fractions. Although direct kinetic evidence for enhanced chain transfer was not obtained in this work, the observed decrease in both *M*_w_ and *M*_z_ is consistent with such an interpretation.

The dispersity (Ð = *M*_w_/*M*_n_) results show that the reference sample has a Ð value of 3.90, corresponding to a relatively narrow molecular weight distribution. In contrast, the Ð values rise to 4.37 and 4.21 for the isopropanol- and n-butanol-modified samples, respectively, indicating a markedly broadened molecular weight distribution. This may be because the alcohol modifiers diversify the types of active centers and enlarge the differences in chain-growth and termination rates among the various active sites; however, direct evidence for such active-center heterogeneity was not obtained in this work. The 2-methoxyethanol-modified sample gives a Ð of 3.94, close to that of the reference sample, exhibiting a favorable uniformity of the molecular weight distribution.

Combined with the foregoing DSC data, it can be found that the highest melting temperature of the reference sample matches its maximum molecular weight. Although the isopropanol-modified sample possesses the optimal isotacticity, its molecular weight decreases significantly, which may offset the positive effect of high stereoregularity and contribute to the lower melting point compared with that of the reference sample. Furthermore, the broader molecular weight distribution of the modified samples is expected to improve the melt flowability of the polypropylene, which may be beneficial for the subsequent processing and molding of the material.

## 4. Conclusions

In this work, different alcohol modifiers were introduced during the synthesis of magnesium ethoxide. The crystal and pore structures of the supports were tuned without destroying their macroscopic Structure. The alcohol modifiers regulate the nucleation and growth of magnesium ethoxide through steric-hindrance and coordination effects, while keeping the original crystalline phase unchanged and altering the stacking order of the layered crystals. After titanation with TiCl_4_, mesoporous Ziegler–Natta catalysts with refined MgCl_2_ crystallites were obtained. Although alcohol modification slightly reduces the titanium loading, the optimized pore structure improves the accessibility of active sites. Consequently, all the modified catalysts exhibit higher propylene polymerization activity than the unmodified reference sample. Isopropanol and n-butanol modification improve the isotactic index, bulk density and crystallization capacity of the polypropylene. In contrast, the ether groups in 2-methoxyethanol may impair the stereodirecting ability of the catalysts. The alcohol modifiers appear to facilitate chain-transfer reactions, reducing the average molecular weight of the polypropylene. Moreover, isopropanol and n-butanol broaden the molecular weight distribution, which is beneficial for polymer processability. Benefiting from the particle replication effect, the polypropylene powders from the modified catalysts contain low fine-powder fractions and satisfy the requirements of industrial production. This study provides a feasible strategy for the custom design of magnesium-ethoxide-based Ziegler–Natta catalysts.

## Figures and Tables

**Table 1:** Particle characteristics of Mg(OEt)_2_.

Sample	Alcoholic Modifier	D_50_ (μm)	Bulk Density (g/cm^3^)	Circularity Degree
MGE-A	-	25.10	0.31	0.90
MGE-B	i-propanol	9.43	0.35	0.90
MGE-C	n-butanol	15.83	0.34	0.89
MGE-D	2-methoxyethanol	17.83	0.34	0.87

Reaction conditions: stirring speed 300 r/min, reaction time 3 h, temperature 70 °C, mass ratio of alcohol to magnesium 11:1, volume ratio of alcohol modifier to ethanol 0.034, molar ratio of iodine to magnesium 0.0027:1.

**Table 2:** Pore structure parameters and titanium content of Ziegler–Natta catalysts derived from different magnesium ethoxide supports.

Sample	Titanium Content (wt%)	Surface Area (m^2^·g^−1^)	Pore Volume (cm^3^·g^−1^)	Pore Size (nm)
Cat-Et	3.44	4.40	0.01	10.09
Cat-*i*Pr	3.18	218.81	0.21	3.73
Cat-*n*Bu	3.12	65.19	0.08	4.63
Cat-ME	3.09	188.79	0.21	4.18

**Table 3:** Polymerization performances of catalysts and corresponding polypropylene properties.

Cat.	Ti (wt%)	Activity ^a^ (kg PP·g^−1^ _Cat_)	I.I. ^b^ (%)	BD ^c^ (g/cm^3^)	*T*_m_ (°C)	*T*_c_ (°C)	*χ*_c_ ^d^ (%)
Cat-Et	3.44	18.50 ± 0.5	98.42 ± 0.1	0.38 ± 0.01	166.10	100.00	39.8
Cat-iPr	3.18	20.18 ± 0.5	99.12 ± 0.1	0.48 ± 0.01	164.90	103.70	43.4
Cat-nBu	3.12	21.62 ± 0.5	99.10 ± 0.1	0.42 ± 0.01	163.30	104.40	40.6
Cat-ME	3.09	21.00 ± 0.5	98.03 ± 0.1	0.45 ± 0.01	162.70	93.90	33.8

^a^ Polymerization conditions: Propylene = 500 g, *n*(Al)/*n*(Ti) = 300, Cocatalyst is triethylaluminium, Cat = 10.0 ± 2 mg, *n*(Al)/*n*(Si) = 20, Electron-donating group is cyclohexylmethyl dimethoxysilane, Hydrogen = 0.05 MPa, T = 70 °C, t = 1 h. The activity is reported as the average of three replicate polymerization runs performed with each catalyst under identical conditions. ^b^ The isotactic index of each polypropylene sample was determined in triplicate (three independent n-heptane extractions). ^c^ The bulk density is reported as the average of three measurements performed on the polypropylene powder obtained from each polymerization run. ^d^ The degree of crystallinity was calculated as the ratio of the melting enthalpy of the polymer to that of fully crystalline i-PP, with the theoretical melting enthalpy of fully crystalline i-PP taken as 207 J/g.

**Table 4:** The particle-size distribution results for polypropylene powders prepared with different catalysts.

Polypropylene *	Polymer Particle Distribution (wt%)					
	>1500 μm	833~1500 μm	429~833 μm	250~429 μm	125~250 μm	<125 μm
PP1	21.256	72.714	4.342	0.961	0.573	0.154
PP2	13.827	15.723	18.181	50.640	1.274	0.355
PP3	10.627	20.244	45.833	15.409	7.387	0.500
PP4	8.052	39.490	42.057	6.661	3.328	0.412

* PP1 to PP4 were prepared using Cat-Et, Cat-*i*Pr, Cat-*n*Bu and Cat-ME, respectively.

**Table 5:** Molecular weight and molecular weight distribution study of polymer resins synthesized with different catalysts.

Sample	M_w_ × 10^5^ (g/mol)	M_n_ × 10^5^ (g/mol)	Ð *	M_p_ × 10^5^ (g/mol)	M_z_ × 10^5^ (g/mol)
PP1	5.30	1.36	3.90	3.37	15.47
PP2	4.77	1.09	4.37	3.02	13.15
PP3	4.93	1.17	4.21	2.99	13.47
PP4	4.65	1.18	3.94	2.92	12.39

* The polydispersity index (Ð) is defined as Ð = *M*_w_/*M*_n_.

## Data Availability

The original contributions presented in this study are included in the article. Further inquiries can be directed to the corresponding authors.

## References

[1] Bahri-Laleh N., Hanifpour A., Mirmohammadi S.A., Poater A., Nekoomanesh-Haghighi M., Talarico G., Cavallo L. (2018). Computational modeling of heterogeneous Ziegler-Natta catalysts for olefin polymerization. Prog. Polym. Sci..

[2] Dashti A., Ahmad Ramazani S.A., Hiraoka Y., Kim S.Y., Taniike T., Terano M. (2009). Kinetic and Morphological Investigation on the Magnesium Ethoxide-Based Ziegler-Natta Catalyst for Propylene Polymerization Using Typical External Donors. Macromol. Symp..

[3] Jandaghian M.H., Maddah Y., Nikzinat E., Masoori M., Sepahi A., Rashedi R., Houshmandmoayed S., Davand R., Afzali K. (2021). Investigation of the effect of Mg(OEt)_2_ manipulation on the ethylene and 1-butene co-polymerization performance of ZieglerNatta catalysts. J. Macromol. Sci. A.

[4] Hongmanee G., Chammingkwan P., Taniike T., Terano M. (2016). Probing into morphology evolution of magnesium ethoxide particles as precursor of Ziegler-Natta catalysts. Polyolefins J..

[5] Abedi S., Azadi F., Daftari-Besheli M., Majdabadi-Farahani N., Safinejad A. (2014). Butyl chloride as promoter in ethylene polymerization by magnesium ethoxide-supported catalys. J. Appl. Polym. Sci..

[6] Abazari M., Jamjah R., Bahri-Laleh N., Hanifpour A. (2022). Synthesis and evaluation of a new three-metallic high-performance Ziegler–Natta catalyst for ethylene polymerization: Experimental and computational studies. Polym. Bull..

[7] Mohamadi Z., Moradi G., Teimoury H. (2021). Comparison of Mg-ethoxide based Ziegler Natta catalysts using different internal donors employed for ethylene polymerization. J. Polym. Res..

[8] Piovano A., Wada T., Amodio A., Takasao G., Ikeda T., Zhu D., Terano M., Chammingkwan P., Groppo E., Taniike T. (2021). Formation of Highly Active Ziegler–Natta Catalysts Clarified by a Multifaceted Characterization Approach. ACS Catal..

[9] Tanase S., Sadashima T., Funabashi H., Kuramoto M. (2004). Magnesium Compound, Solid Catalyst Component for Olefin Polymerization, Catalyst for Olefin Polymerization and Method for Producing Polyolefin. Taiwan Patent.

[10] Tsuda T., Ota T., Funabashi H., Tanase S. (2001). Magnesium Compound, Olefin Polymerization Catalyst and Method for Producing Olefin Polymer. Japan Patent.

[11] Beppu A., Kobayashi H. (2006). Production of Spherical Fine Particulate Magnesium Ethylate. Japan Patent.

[12] Chauhan S., Shah S., Patil H.R., Gupta V. (2021). Investigating effect of mixed alcohols and in-situ initiator on magnesium alkoxide for Ziegler–Natta catalyst and polypropylene improvement. J. Polym. Res..

[13] Wada T., Funako T., Chammingkwan P., Thakur A., Matta A., Terano M., Taniike T. (2020). Structure-performance relationship of Mg(OEt)_2_-based Ziegler-Natta catalysts. J. Catal..

[14] Kang K.K., Kim K.-S., Lee D.-H., Jeong Y.-T. (2001). Propylene Polymerization by Using TiCl_4_ Catalyst Supported on Solvent-Activated Mg(OEt)_2_. J. Appl. Polym. Sci..

[15] Yabunouchi N., Sadashim T. (2004). Catalysts for Polymerizing Olefins and Process for Producing Olefin Polymer. WIPO Patent.

[16] Hamedani N.G., Behrouzi-Fardmoghadam M., Abedini H., Bahri-Laleh N., Poater A. (2025). Optimization of Starting Material Composition in the Synthesis of Highly Active Magnesium Ethoxide-Supported Ziegler–Natta Catalysts for Propylene Polymerization. J. Polym. Sci..

[17] Chammingkwan P., Khoshsefat M., Terano M., Taniike T. (2023). Parallel catalyst synthesis protocol for accelerating heterogeneous olefin polymerization research. Polymers.

[18] Zohuri G.H., Kasaeian A.B., Angagi M.T., Jamjah R., Mousavian M.A., Emami M., Ahmadjo S. (2005). Polymerization of propylene using MgCl_2_(ethoxide type)/TiCl_4_/diether heterogeneous Ziegler–Natta catalyst. Polym. Int..

[19] Tanase S., Katayama K., Inasawa S., Okada F., Yamaguchi Y., Konakazawa T., Junke T., Ishihara N. (2008). Particle growth of magnesium alkoxide as a carrier material for polypropylene polymerization catalyst. Appl. Catal. A.

[20] Ren H., Qiu Y., Yao Q., Yang B. (2026). Research on the controlled synthesis of magnesium ethoxide support for Ziegler-Natta catalysts in olefin polymerization. Colloid Polym. Sci..

[21] Joseph J., Singh S.C., Gupta V.K. (2009). Morphology controlled magnesium ethoxide: Influence of reaction parameters and magnesium characteristics. Part Sci. Technol..

[22] Funako T., Chammingkwan P., Taniike T., Terano M. (2015). Alternation of Pore Architecture of Ziegler–Natta Catalysts through Modification of Magnesium Ethoxide. Macromol. React. Eng..

[23] Funako T., Chammingkwan P., Taniike T., Terano M. (2015). Addition of a second alcohol in magnesium ethoxide synthesis as a way to vary the pore architecture of Ziegler-Natta catalysts. Polyolefins J..

[24] Tanase S., Katayama K., Inasawa S., Okada F., Yamaguchi Y., Sadashima T., Yabunouchi N., Konakazawa T., Junke T., Ishihara N. (2008). New Synthesis Method Using Magnesium Alkoxides as Carrier Materials for Ziegler-Natta Catalysts with Spherical Morphology. Macromol. React. Eng..

[25] Behrouzi Fardmoghadam M., Kharat A.N., Shakeri A. (2020). Modified mesoporous magnesium ethoxide with spherical morphology for polypropylene Ziegler–Natta catalyst: Preparation and characterization. J. Inorg. Organomet. Polym. Mater..

[26] Chammingkwan P., Wannaborworn M., Le T.T.M., Terano M., Taniike T., Phiriyawirut P. (2021). Particle engineering of magnesium ethoxide-based Ziegler-Natta catalyst through post-modification of magnesium ethoxide. Appl. Catal. A.

[27] Lan Z., Lu Y. (2020). Tailoring morphology and bulk density of magnesium ethoxideparticles by adding n-hexane and silicone oil. Particuology.

[28] Chammingkwan P., Terano M., Taniike T. (2017). High-throughput synthesis of support materials for olefin polymerization catalyst. ACS Comb. Sci..

[29] (2009). Determination of Bulk Density for Inorganic Chemical Products.

[30] (2023). Plastics—Determination of Apparent Density of Material That Can Be Poured from a Specified Funnel.

